# Growth suppressive effect of pegylated arginase in malignant pleural mesothelioma xenografts

**DOI:** 10.1186/s12931-017-0564-3

**Published:** 2017-05-02

**Authors:** Sze-Kwan Lam, Yuan-Yuan Li, Shi Xu, Leanne Lee Leung, Kin-Pong U, Yan-Fang Zheng, Paul Ning-Man Cheng, James Chung-Man Ho

**Affiliations:** 1 Division of Respiratory Medicine, Department of Medicine, The University of Hong Kong, Queen Mary Hospital, Pokfulam, SAR Hong Kong; 2 Bio-cancer Treatment International Limited, 511-513, Bioinformatics Building, Hong Kong Science Park, Tai Po, Hong Kong, SAR China; 3 Oncology Center, Zhujiang Hospital of Southern Medical University, Guangzhou, China

**Keywords:** Mesothelioma, Pegylated arginase, BCT-100, Apoptosis, Cell cycle arrest, Xenografts

## Abstract

**Background:**

Malignant pleural mesothelioma (MPM) is a difficult-to-treat global disease. Pegylated arginase (BCT-100) has recently shown anti-tumor effects in hepatocellular carcinoma, acute myeloid leukemia and melanoma. This study aims to investigate the effects of PEG-BCT-100 in MPM.

**Methods:**

A panel of 5 mesothelioma cell lines (H28, 211H, H226, H2052 and H2452) was used to study the in vitro effects of BCT-100 by crystal violet staining. The in vivo effects of BCT-100 were studied using 211H and H226 nude mice xenografts. Protein expression (argininosuccinate synthetase, ornithine transcarbamylase, cleaved PARP, cleaved caspase 3, cyclins (A2, D3, E1 and H), CDK4 and Ki67) and arginine concentration were evaluated by Western blot and ELISA respectively. Cellular localization of BCT-100 was detected by immunohistochemistry and immunoflorescence. TUNEL assay was used to identify cellular apoptotic events.

**Results:**

Argininosuccinate synthetase was expressed in H28, H226, and H2452 cells as well as 211H and H266 xenografts. Ornithine transcarbamylase was undetectable in all cell lines and xenograft models. BCT-100 reduced in vitro cell viability (IC_50_ values at 13–24 mU/ml, 72 h) across different cell lines and suppressed tumor growth in both 211H and H226 xenograft models. BCT-100 (60 mg/kg) significantly suppressed tumor growth (*p* < 0.01) with prolonged median survival (*p* < 0.01) in both xenograft models. Combining BCT-100 with pemetrexed or cisplatin conferred no additional benefits over single agents. Serum and intratumoral arginine levels were effectively decreased by BCT-100, associated with cytosolic accumulation of BCT-100 within tumor cells. Apoptosis (PARP cleavage in 211H xenografts; Bcl-2 downregulation, and cleavage of PARP and caspase 3 in H226 xenografts; positive TUNEL staining in both) and G1 arrest (downregulation of cyclin A2, D3, E1 and CDK4 in 211H xenografts; suppression of cyclin A2, E1, H and CDK4 in H226 xenografts) were evident with BCT-100 treatment. Furthermore, proliferative factor Ki67 was downregulated in BCT-100 treatments arms.

**Conclusions:**

BCT-100 suppressed tumor growth with prolonged median survival partially mediated by intratumoral arginine depletion resulting in apoptosis and G1 arrest in mesothelioma xenograft models. The findings provide scientific evidence to support further clinical development of BCT-100 in treatment of MPM.

## Background

Malignant pleural mesothelioma (MPM) arises from exposure to asbestos fibers with a long latency period, which has been a global health problem for the past few decades. Its anticipated peak incidence is yet to be reached. Mortality and morbidity of MPM remain extremely high, as its treatment is rarely curative. Surgery alone or in combination with radiotherapy and chemotherapy [1] remains the standard treatment for early-stage disease. Combination chemotherapy for advanced disease carries only modest survival benefit [2]. Proven effective treatment options are very limited, especially for patients with a poor response to previous chemotherapy.

Amino acids are essential for different cellular functions including synthesis of protein and polyamines [3]. Intuitively, amino acid depletion may serve as an effective treatment for those cancers that are highly dependent on exogenous source of amino acids. Arginine is a semi-essential amino acid in selected tumors, but can be replenished intracellularly in normal cells. Therefore arginine-degrading enzymes (arginase and arginine deiminase (ADI)) have been tested in treating different cancers that are unable to synthesize arginine [4, 5]. Overexpression of argininosuccinate synthetase (ASS1) and ornithine transcarbamylase (OTC) is one of the resistance mechanisms in arginase treatment.

BCT-100 is a pegylated arginase (US FDA IND granted in March 2012) (Bio-Cancer Treatment International Limited, Hong Kong), which has been tested in a phase I/II clinical trial for treatment of hepatocellular carcinoma (HCC) with encouraging results [6]. In vitro, BCT-100 has shown anti-tumor effects in HCC [4, 7] and acute myeloid leukemia (AML) [8] with IC_50_ values at 100–250 mU/ml and 100–1250 μU/ml respectively. Arginase exerts antiproliferative effects in melanoma [9] and HCC [10] by inducing cell cycle arrest and apoptosis. Interestingly an early phase clinical trial of arginine depletion with pegylated ADI (ADI-PEG20) in MPM was reported in 2014 [11]. Among those MPM with deficient ASS1 expression by immunohistochemistry, treatment with ADI-PEG20 achieved good metabolic response with significant improvement in progression-free survival (hazard ratio 0.53, 95% CI: 0.31 to 0.90, *p* = 0.02) compared with best supportive care alone. However, potentially serious anaphylactoid reaction to ADI has been reported. Nonetheless, BCT-100 has been shown in early phase clinical trials to be very well tolerated without anaphylaxis. So far, the effect of BCT-100, with a different mechanism of arginine depletion from ADI, has not been investigated in MPM. We therefore embarked on this study to evaluate the anti-cancer effects of BCT-100 in MPM, which could provide a scientific basis for future clinical development of BCT-100 as a novel treatment for MPM.

## Methods

### Cell lines and reagents

A panel of 5 mesothelioma cell lines (NCI-H28 (sarcomatoid), MSTO-211H (biphasic), NCI-H226 (epithelioid), NCI-H2052 (sarcomatoid) and NCI-H2452 (epithelioid)) was purchased and authenticated (American Type Culture Collection, Manassas, VA, USA). Cells were incubated in RPMI-1640 medium (Gibco®, Life Technologies, Carlsbad, California, USA) enriched with 10% fetal bovine serum (FBS) (Gibco®) in a humidified atmosphere of 5% CO_2_ at 37 °C.

### Pegylated arginase (BCT-100)

Pegylated arginase (BCT-100, PEG-BCT-100 or rhArg1peg5000) was provided by Bio-cancer Treatment International Limited.

### Cell viability assay

Briefly, cells (5000/well) were incubated with different activity units of BCT-100 with/without cisplatin or pemetrexed. Cells incubated with medium only served as a negative control. Following incubation for 72 h, cells were fixed for 10 min with 4% formaldehyde (Sigma-Aldrich, St. Louis, Missouri, United States) in phosphate-buffered saline (PBS) and stained for 10 min with 0.05% crystal violet. The plates were washed for 4 times with tap water and then the cells were lysed with 1% sodium dodecyl sulfate (SDS) solution (Sigma-Aldrich). Absorbance (595 nm) was measured using a microplate reader Fluo Star Optima (Bmg Labtec GmbH, Ortenberg, Germany) [12].

### Tumor growth inhibition in vivo

The 211H and H226 xenograft models were created, while H28, H2052 and H2452 xenografts could not be developed successfully, by subcutaneous injection of 10^7^ corresponding cells in PBS into the upper back of 18 nude mice (female, 4–6-week-old, 10–14 g, BALB/cAnN-nu, Charles River Laboratories, Wilmington, USA). Mice were randomized into 3 groups after tumor growth was established. PBS (served as control), BCT-100 (20 and 60 mg/kg twice a week), pemetrexed (Eli Lilly, Indianapolis, Indiana, USA) (150 mg/kg twice a week) and/or cisplatin (Cayman, Teaduspargi, Tallinn, Estonia) (2.5 mg/kg once/week) were administered intraperitoneally. Tumor dimension (using standard calipers) and body weight of mice were measured twice a week and tumor volume was calculated [volume = length x width x width)/2] [13]. For humane reasons, mice were sacrificed when tumor volume reached 600 mm^3^. Tumor xenografts were collected. The study protocol was approved by the institutional Animal Ethics Committee (approval reference number: CULATR 3463-14), and standard humane endpoints for animal research were applied.

### Study of protein expression with Western blot, immunohistochemistry and immunofluorescence staining

Western blot was performed as previously described [12]. Specific primary antibodies [mouse monoclonal anti-human β-actin (Sigma-Aldrich), anti-ASS1, anti-OTC, anti-PARP, (Santa Cruz Biotechnology, Inc., Santa Cruz, California, USA), anti-Bcl-2, anti-cleaved caspase-3, anti-cyclin A2, D3, E1, H, anti-CDK4 (Cell Signaling Technology, Danvers, Massachusetts, USA), anti-Ki67 (ImmunoWay Biotechnology Company, Texas, USA), anti-PEG (RevMAb, San Francisco, USA) antibodies] and corresponding horseradish peroxidase (HRP)-conjugated secondary (Cell Signaling Technology) were purchased. An enhanced chemiluminescence (ECL) kit (GE Healthcare) was used to detect protein expression. Beta-actin was selected as reference protein [14].

Immunohistochemistry and immunofluorescence staining were carried out according to standard protocol. Anti-cytokeratin 1 (Santa Cruz Biotechnology), anti-PEG and corresponding Alexa Fluor anti-mouse or anti-rabbit (Life Technology) antibody were used. The slides were mounted with Prolong® Gold antifade reagent with 4′,6-diamidino-2-phenylindole (DAPI, Life Technologies). Photos were taken with Nikon Ni-U fluorescence microscope (Nikon, Tokyo, Japan) equipped with camera/detector Diagnostic Instrument RT3 Slider (Meyer Instruments, Houston, USA).

### Serum arginine concentration detection

L-arginine ELISA kit was purchased from Immundiagnostik (Bensheim, Hessen, Germany). The procedure was carried out according to manufacturer’s protocol. Briefly, derivatized control, standards and samples were incubated with L-arginine antibody overnight. Peroxidase conjugate was added after washing. Stop solution was then added to cease reaction after incubation with tetramethybenzidine substrate. Absorbance (450 nm) was measured with a reference (620 nm) using a microplate reader Fluo Star Optima.

### Terminal Deoxynucleotidyl transferase-dUTP Nick End Labeling (TUNEL) Assay

TUNEL assay was performed according to standard protocol using Click-iT® Plus TUNEL Assay (Life Technologies). Formalin-fixed, paraffin embedded tumor xenograft sections were de-paraffinized, fixed and permeablized. Sections were first incubated with terminal deoxynucleotidyl transferase (TdT) reaction buffer, then TdT buffer containing EdUTP, TdT and TdT enzyme. Slides were stained with TUNEL reaction cocktail (TUNEL reaction buffer, copper protectant, Alexa Fluor® picoyl azide and TUNEL reaction buffer additive). The slides were mounted with Prolong® Gold antifade reagent with DAPI. Photos were taken with Nikon Ni-U fluorescence microscope.

### Statistical analysis

Experiments were repeated at least 3 times and data analysed (mean ± standard deviation). The difference between groups was analysed using Student’s two-tailed *t*-test by Prism (GraphPad Software, La Jolla, Southern California, USA). A *p*-value < 0.05 defined statistical significance (*: *p* < 0.05, **: *p* < 0.01, ***: *p* < 0.001). Survival time of mice bearing tumor xenografts was defined as the time lapse between the start of treatment and time point when humane endpoints were reached. The difference of median survival between treatment arms was analysed using log-rank test by Prism.

## Results

### In vitro activity of BCT-100 in mesothelioma

**Fig. 1:**
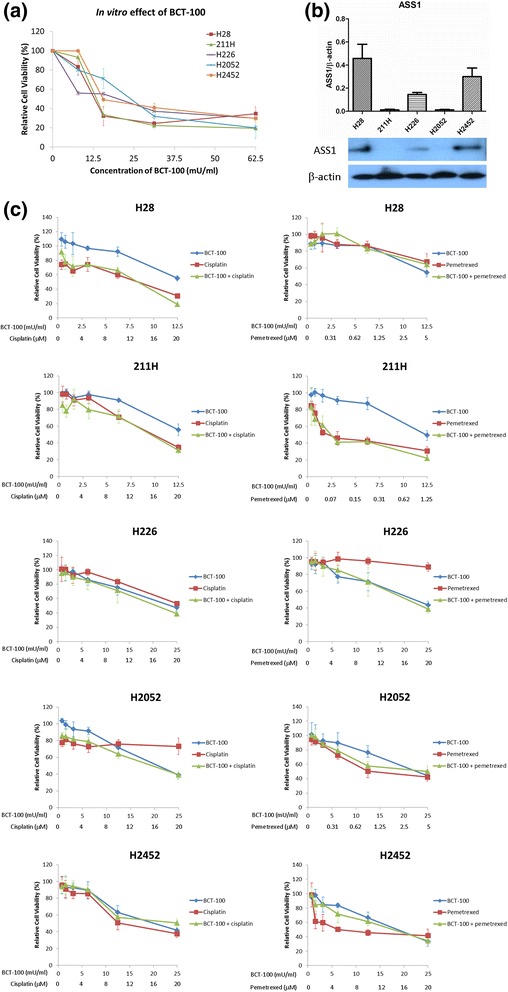
Relative cell viability upon BCT-treatment, basal expression of ASS1 and combination effect of BCT-100 with cisplatin or pemetrexed in a panel of mesothelioma cell lines. **a** BCT-100 reduced cell viability in a dose-dependent fashion. **b** ASS1 was found in H28, H226 and 2452 cells but not in 211H and H2052 cells. **c** No synergistic effect in combination of BCT-100 with cisplatin or pemetrexed in different mesothelioma cell lines

Treatment with BCT-100 induced a dose-dependent antiproliferative effect in all mesothelioma cell lines. The IC_50_ values in H28, 211H, H226, H2052 and H2452 cells were 14.3 ± 2.0, 13.0 ± 1.8, 22.4 ± 6.0, 20.8 ± 5.6 and 18.6 ± 6.5 mU/ml respectively after 72 h of treatment (Fig. 1a).

### Basal in vitro expression of ASS1 and OTC

ASS1 was highly expressed in H28 and H2452 cells, moderately expressed in H226 cells while not detectable in 211H and H2052 cells (Fig. 1b). All cell lines were OTC negative (data not shown). As both ASS1 and OTC are critical rate-limiting enzymes in the urea cycle responsible for replenishing intracellular arginine, depletion of either one of them is sufficient for cellular dependence on exogenous arginine supplementation. This could possibly explain why BCT-100 was uniformly effective despite varying ASS1 expression across our panel of MPM cell lines.

### Combination of BCT-100 with cisplatin or pemetrexed

There was no synergism when combining BCT-100 with cisplatin or pemetrexed in all cell lines (Fig. 1c)

### Tumor xenograft growth suppression with BCT-100

**Fig. 2:**
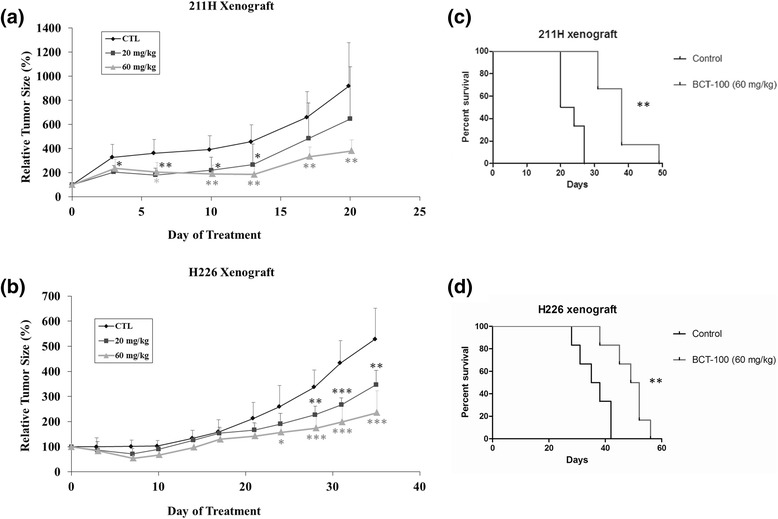
BCT-100 suppressed tumor growth and increased median survival in MPM xenograft models. BCT-100 inhibited tumor growth in a dose-dependent manner in (**a**) 211H and (**b**) H226 xenografts. BCT-100 (60 mg/kg) increased median survival in (**c**) 211H and (**d**) H226 xenograft models

After inoculation of 211H or H226 cells for 3 days, obvious tumors were established. In 211H xenograft, after 20 days of BCT-100 treatment, the relative tumor volume in the 20 and 60 mg/kg treatment groups were 70.5 and 41.4% (*p* < 0.01) that of the control group respectively (Fig. 2a). In H226 xenograft, after 35 days of BCT-100 treatment, the relative tumor volume in the 20 and 60 mg/kg treatment groups were 65.5% (*p* < 0.01) and 44.4% (*p* < 0.001) that of the control group respectively (Fig. 2b).

### Prolonged median survival with BCT-100 in xenograft models

The median survival (from start of treatment till reaching humane endpoints) increased from 22 days in control arm to 38 days in 60 mg/kg BCT-100 treatment arm (*p* < 0.01) in 211H xenograft model (Fig. 2c). Similarly, the median survival increased from 36.5 days in control group to 50.5 days in 60 mg/kg BCT-100 treatment arm (*p* < 0.01) in H226 xenograft (Fig. 2d).

### Combination effects of BCT-100 with pemetrexed or cisplatin on tumor xenografts

**Fig. 3:**
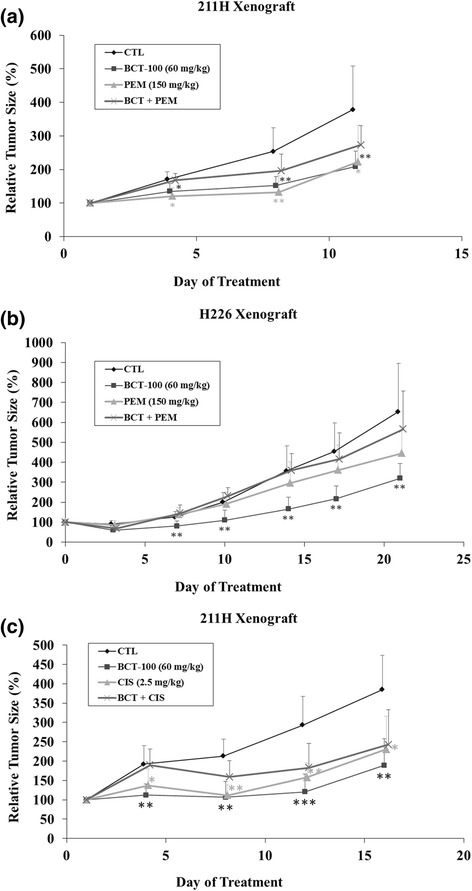
Combination effects of BCT-100 and pemetrexed or cisplatin in MPM xenograft models. No synergistic or additive effect was observed when combining BCT-100 with pemetrexed in (**a**) 211H and (**b**) H226 xenograft models. The tumor suppressive effect of BCT-100 was comparable to pemetrexed in 211H xenograft model. Pemetrexed exerted no tumor suppression in H266 xenografts. **c** There was no beneficial effect when combining BCT-100 with cisplatin in 211H xenograft model

No synergistic effect was observed when combining BCT-100 with pemetrexed in both xenografts. In 211H xenograft model, both BCT-100 and pemextrexed exerted similar tumor suppressive effect (Fig. 3a). In H226 xenograft model, only BCT-100 showed significant tumor suppression but not pemetrexed (Fig. 3b).

In 211H xenograft model, no beneficial effect was noted when combining BCT-100 with cisplatin (Fig. 3c). More importantly, liver cirrhosis was observed in all mice in BCT-100/cisplatin arms. Therefore, such combination was not pursued in H226 xenograft model.

### Basal in vivo expression of ASS1 and OTC

**Fig. 4:**
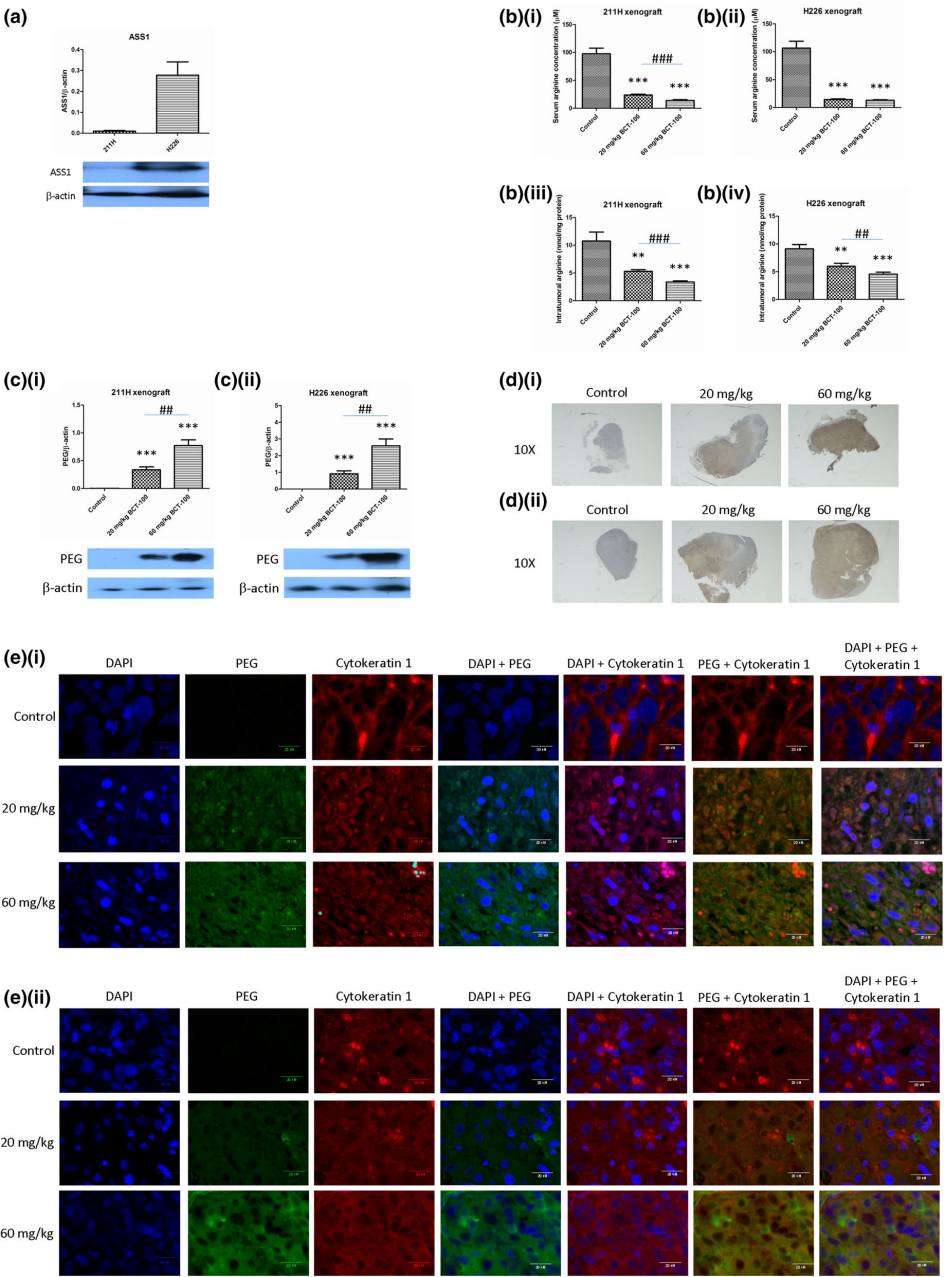
Basal expression of ASS1, BCT-100-induced arginine depletion and localization of BCT-100 in xenograft models. **a** ASS1 was highly expressed in H226 xenograft but relatively low in 211H xenograft. BCT-100 decreased serum arginine concentration in (**b**)(**i**) 211H and (**b**)(**ii**) H226 xenograft models. BCT-100 reduced intratumoral arginine level in a dose-dependent fashion in (**b**)(**iii**) 211H and (**b**)(**iv**) H226 xenografts. Intratumoral level of PEG-BCT-100 was increased in BCT-100 treatment arms with a dose-dependent manner in (**c**)(**i**) 211H and (**c**)(**ii**) H226 xenografts. BCT-100 was mostly located in the central part of tumor in (**d**)(**i**) 211H and (**d**)(**ii**) H226 xenografts, with cytosolic compartmentalization ((**e**)(**i**), (**e**)(**ii**)) as shown by immunoflorescence staining (pictures taken with 400X magnification)

ASS1 was highly expressed in H226 xenograft while only weakly detected in 211H xenograft (Fig. 4a). Both xenografts were OTC negative (data not shown).

### In vivo arginine depletion by BCT-100

The serum arginine concentration in control group was 98 ± 36 μM in both xenograft models. In 211H xenograft, the serum arginine concentration decreased to 24 ± 5 and 14 ± 5 μM in 20 and 60 mg/kg BCT-100 treatment groups respectively (*p* < 0.001), with significant dose-dependent difference between treatment arms (*p* < 0.001) (Fig. 4b(i)). In H226 xenograft, the serum arginine concentration also reduced to 14 ± 4 and 13 ± 4 μM in 20 and 60 mg/kg BCT-100 treatment groups respectively (*p* < 0.001) (Fig. 4b(ii)), though there was no significant between-group difference.

In 211H xenograft, intratumoral arginine content decreased from 9.7 ± 2.3 nmole/mg protein in the control arm to 5.3 ± 1.1 nmole/mg protein (20 mg/kg BCT-100 arm, *p* < 0.001) and 3.4 ± 0.7 nmole/mg protein (60 mg/kg BCT-100 arm, *p* < 0.001). There was significant dose-dependent intratumoral arginine depletion among BCT-100 treatment groups (*p* < 0.001) (Fig. 4b(iii)). In H226 xenograft, intratumoral arginine content declined from 9.1 ± 2.5 nmole/mg protein in the control group to 6.2 ± 1.8 nmole/mg protein (20 mg/kg BCT-100 arm, *p* < 0.001) and 4.3 ± 1.3 nmole/mg protein (60 mg/kg BCT-100 arm, *p* < 0.001). There was also significant dose-dependent intratumoral arginine depletion between 20 and 60 mg/kg BCT-100 groups (*p* < 0.01) (Fig. 4b(iv)).

### Penetration of BCT-100 into tumor xenografts

Since BCT-100 was linked to polyethylene glycol (PEG), anti-PEG antibody was used to identify the location of BCT-100. Intratumoral BCT-100 was found in BCT-100 treatment arms in 211H (Fig. 4c(i)) and H226 (Fig. 4c(ii)) xenograft models in a dose-dependent manner. BCT-100 was found mostly in the central part of tumor xenografts as shown by immunohistochemistry (IHC) in 211H (Fig. 4d(i)) and H226 (Fig. 4d(ii)) xenograft models.

The compartmentalization of BCT-100 was determined using immunofluorescence staining. DAPI (blue color) and cytokeratin 1 (red color) were used to identify the location of nucleus and cytoplasm respectively. PEG staining (green color) was shown to overlap mainly with cytokeratin 1 staining in both 211H (Fig. 4e(i)) and H226 (Fig. 4e(ii)) xenograft models.

### Apoptosis induced by BCT-100 in xenograft models

**Fig. 5:**
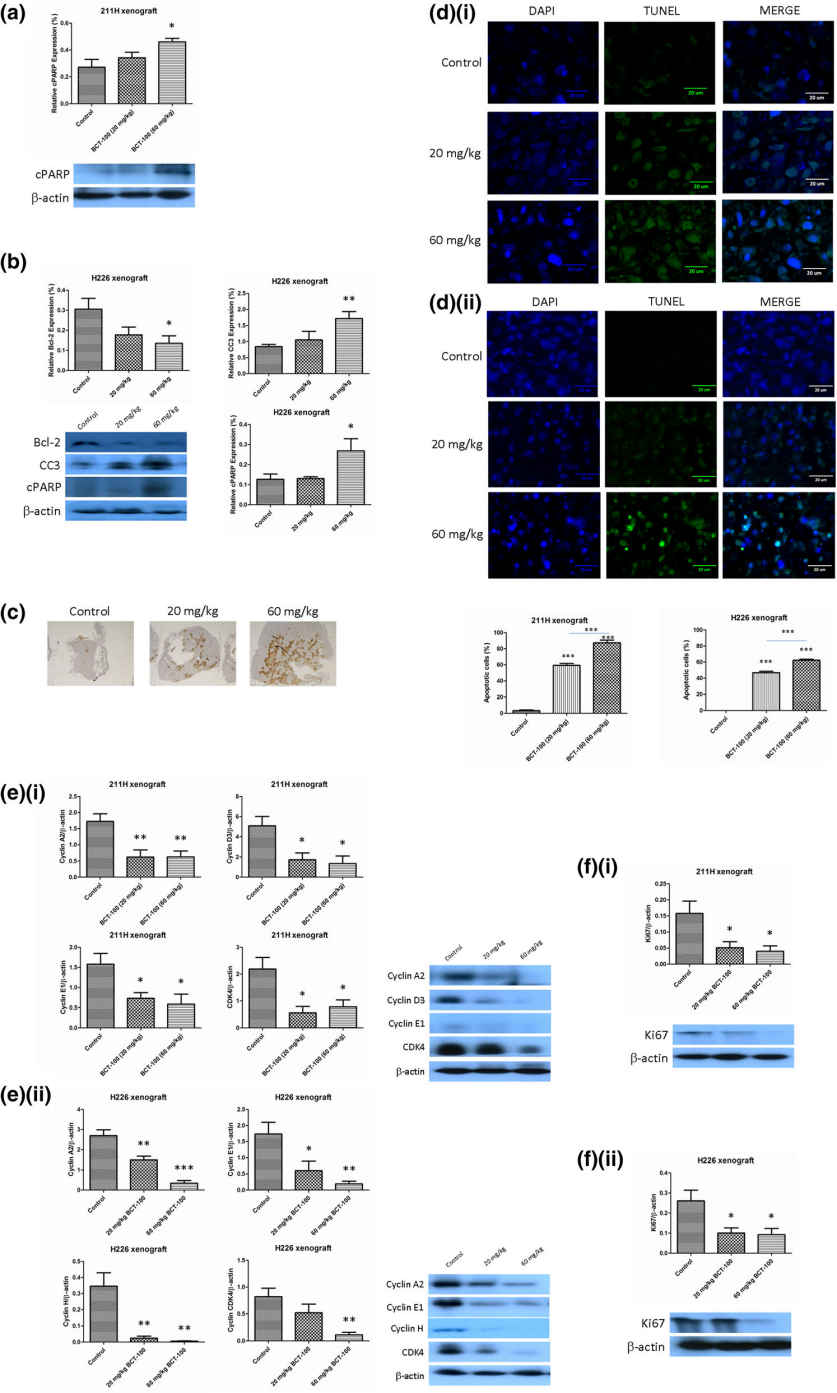
BCT-100 induced apoptosis and G1 arrest in MPM xenografts. Upon BCT-100 (60 mg/kg) treatment, (**a**) cleaved PARP (cPARP) was upregulated in 211H xenograft, (**b**) downregulation of Bcl-2 as well as cleavage of caspase 3 (CC3) and PARP (cPARP) in H226 xenograft and (**c**) CC3 was mainly found in the core part of tumor in H226 xenograft. (**d**) Nuclei were stained by DAPI (*blue signal*). There was no green TUNEL signal in control groups in both xenografts. The TUNEL signal was noted in both treatment arms in (**d**)(**i**) 211H and (**d**)(**ii**) H226 xenografts. Overlay of TUNEL and DAPI indicated DNA breaks were mainly located in nuclei. **e**(**i**) BCT-100 reduced the expression of cyclin A2, D3, E1 and CDK4 in 211H xenograft. **e**(**ii**) Downregulation of cyclin A2, E1, H and CDK4 by BCT-100 was observed in H226 xenograft. BCT-100 induced downregulation of proliferation factor Ki67 in (**f**)(**i**) 211H and (**f**)(**ii**) H226 xenografts

PARP cleavage was observed in 60 mg/kg BCT-100 treatment arm in 211H xenograft (Fig. 5a). Downregulation of Bcl-2 as well as upregulation of cleaved caspase 3 (CC3) and cleaved PARP (cPARP) were also noted in 60 mg/kg BCT-100 treatment group in H226 xenograft (Fig. 5b). CC3 was mainly located in the central part of tumor xenograft as shown by IHC staining in H226 xenograft (Fig. 5c).

In TUNEL assay, TUNEL-positive DNA strand breaks were noted in BCT treatment arms in 211H (Fig. 5d(i)) and H226 (Fig. 5d(ii)) xenografts.

### G1 arrest induced by BCT-100 in vivo

G1 arrest was indirect evidenced by alteration of related protein expression in cell cycle. Cyclin A2, D3, E1 and CDK4 were downregulated by BCT-100 in 211H xenograft (Fig. 5e(i)) while cyclin A2, E1, H and CDK4 were suppressed by BCT-100 in H226 xenograft (Fig. 5e(ii)). Expression of Ki67 was suppressed by BCT-100 in both 211H (Fig. 5f(i)) and H226 (Fig. 5f(ii)) xenograft models.

## Discussion

BCT-100 showed antiproliferative effects in MPM as demonstrated by crystal violet cell viability assay in our cell line model. In 211H and H226 xenograft models, BCT-100 (60 mg/kg) suppressed tumor growth accompanied by prolonged median survival. Serum arginine concentration and intratumoral arginine content decreased significantly in BCT-100 treatment groups, associated with apoptosis and G1 arrest.

Amino acid depletion is a novel approach for cancer treatment. Asparagine and arginine are both essential amino acids in cancer cells, but not in normal human cells. ADI and BCT-100 were pre-clinically and clinically proven to inhibit arginine auxotrophic tumors with ADI targeting ASS1 deficient cancers and BCT-100 targeting ASS1 and/or OTC negative cancers. Furthermore, ADI-PEG20 has been shown to have interesting anti-cancer effect on ASS-deficient cancers including lymphoma, head and neck cancer, small cell lung cancer, pancreatic cancer and breast cancer through apoptosis and cell cycle arrest [15].

Arginine depletion carries a systemic effect that may involve the tumor microenvironment. Therefore, we believe that the effect of BCT-100 would be better studied using xenograft models rather cell line models. Since the in vivo tumor suppressive effect of BCT-100 alone has been observed in our study, the combination of BCT-100 and current active chemotherapeutic agents (pemetrexed or cisplatin) in MPM was further investigated. In 211H xenograft model, the effect of BCT-100 was similar to pemetrexed or cisplatin. More importantly, BCT-100 showed a superior effect than pemetrexed in H226 xenograft. It has been shown that thymidylate synthase was highly expressed in H226 xenograft [14] which can explain the relative pemetrexed resistance in H226 xenograft. Nonetheless, the combination of BCT-100 and pemetrexed did not reveal any additional benefit compared with single agents. As serious side effect (liver cirrhosis) was found with BCT-100/cisplatin combination in 211H xenograft model, this combination was not pursued further in our study.

The common side effects of cisplatin and pemetrexed combination chemotherapy include severe nausea and vomiting [16], myelosuppression and neutropenia [17]. With good tolerability of BCT-100 shown in early clinical trials [6], BCT-100 may potentially serve as an alternative therapeutic agent, though not in combination with existing chemotherapy, in the clinical management of MPM. This is particularly true among patients with MPM that overexpress thymidylate synthase resulting in pemetrexed resistance.

MPM can be sub-divided into epithelioid, sarcomatoid and biphasic histological sub-types, with poorer survival among patients with sarcomatoid and biphasic mesothelioma [18]. BCT-100 showed tumor suppressive effect in biphasic 211H and epithelioid H226 MPM xenograft models. However, no sarcomatoid mesothelioma xenograft model could be developed in this study for assessment of the efficacy of BCT-100.

BCT-100 effectively decreased serum arginine concentration to 24 and 14% that of control mice in both xenograft models even with low-dose (20 mg/kg) BCT-100 treatment, which could not completely explain the dose-dependent tumor suppression. On the other hand, the dose-dependent arginine depletion was better demonstrated within the intratumoral compartment, which should be more relevant to the activity of BCT-100 on the tumor xenografts. This is in line with the finding of increased intratumoral BCT-100 level with higher dose of BCT-100 treatment, suggesting intratumoral on top of systemic arginine depletion. The intratumoral localization of BCT-100 in MPM xenografts was mainly cytosolic, which is similar to previous finding [19].

Apoptotic cells were increased after incubating melanoma A375 cells with BCT-100 using annexin-V binding assay [9]. Caspase-dependent cellular apoptosis was activated by BCT-100 in human hepatoma HepG2 and PLC/PRF/5 cells [4]. Nonetheless, the in vivo evidence of apoptosis has not yet been shown previously. In our study, apoptosis was induced by BCT-100 in mesothelioma xenograft models as evidenced by alteration of key apoptosis-related factors and TUNEL positive-staining in BCT-100 treatment arms in both xenografts.

It has been shown that BCT-100 could induce G2/M arrest in HCC Hep3B cells, S arrest in HCC HepG2 and PLC/PRF/5 cells [10], and G2/M arrest in melanoma A375 cells [9]. On the other hand, G0/G1 arrest was observed with BCT-100 treatment in acute myeloid leukemia cells [19]. BCT-100-induced G1 arrest was evidenced by altered expression of relevant cell cycle regulators in our in vivo mesothelioma study, suggesting that the exact point of cell cycle arrest could be cell line- or tumor-specific. Ki67 is a functional antigen found in the nucleus that serves as a proliferative marker controlling all phases of the cell cycle [20]. Downregulation of Ki67 by BCT-100 in both MPM xenografts is consistent with suppression of G1 arrest-related factors.

BCT-100 reduced relative cell viability in Hep3B (hepatoma), A375 (melanoma), 211H (mesothelioma) and H226 (mesothelioma) cells with IC_50_ values of 100, 86, 13 and 20 mU/ml respectively [4, 9]. The effective dose of BCT-100 used was about 27000 U/kg/week in both melanoma and HCC xenograft models [4, 9] while only about 3000 U/kg/week was administered in MPM xenograft models in our study. The correlation between in vitro and in vivo efficacy of BCT-100 seems to be quite consistent among different tumor types. Furthermore, the dosage of BCT-100 given in a clinical trial for treatment of advanced HCC was 1600 U/kg/week [6], which was equivalent to about 20000 U/kg/week in mice models [21]. The effective dose of BCT-100 used in this study was only 3000 U/kg/week which was much lower than that the equivalent dose used in the clinical trial. The dose of BCT-100 used was likely cancer-specific which was possibly due to different types of cancer cells having different arginine and metabolic requirements [6]. Our finding of the relatively greater sensitivity of MPM than HCC or melanoma to BCT-100 holds promise for future clinical development of BCT-100 in the treatment of MPM. Ideally, the efficacy of BCT-100 in MPM can be better evaluated in primary cultures that provide closer mimicry of tumor microenvironment. However, MPM is a rare disease in Hong Kong with only about 20 new cases per year, which precludes any feasibility of experiments using primary cultures derived from patients’ tumor samples.

Recently, an early-phase clinical trial was conducted using pegylated arginase (BCT-100) at 1600 U/kg/week in the treatment of advanced HCC [6]. A significantly improved progression-free survival was observed in patients with adequate arginine depletion (ADD) (<8 μM serum arginine concentration) for more than 2 months compared with those with ADD for less than 2 months (6.4 vs 1.7 months, 95% CI: 1.67–1.73, *p* = 0.01). Overall survival was also improved with BCT-100 treatment compared with untreated HCC patients (6.4 vs <2–3 months [22]). Adverse events were mostly mild (pain, insomnia, loss of appetite, constipation, fatigue, vomiting and nausea) with BCT-100 treatment [6]. Further clinical development of BCT-100 in treatment of unresectable HCC is currently underway.

## Conclusion

BCT-100 as a single agent has demonstrated significant in vitro and in vivo anti-tumor effects in MPM, which are mediated by intratumoral arginine depletion resulting in apoptosis and cell cycle arrest.

## Abbreviations

ADI: Arginine deiminase
ASS1: Argininosuccinate synthetase
CC3: Cleaved caspase 3
cPARP: Cleaved PARP
DAPI: 4′,6-diamidino-2-phenylindole
HCC: Hepatocellular carcinoma
MPM: Malignant pleural mesothelioma
OTC: Ornithine transcarbamylase
TUNEL: Terminal Deoxynucleotidyl transferase-dUTP Nick End Labeling
